# Long-term outcomes of primary aortic valve repair in children with congenital aortic stenosis – 15-year experience at a single center

**DOI:** 10.3389/fcvm.2022.1029245

**Published:** 2022-10-13

**Authors:** Qiushi Ren, Juemin Yu, Tianyu Chen, Hailong Qiu, Erchao Ji, Tao Liu, Xiaowei Xu, Jianzheng Cen, Shusheng Wen, Jian Zhuang, Xiaobing Liu

**Affiliations:** ^1^Department of Cardiovascular Surgery, Guangdong Cardiovascular Institute, Guangdong Provincial People’s Hospital, Guangdong Academy of Medical Sciences, Guangzhou, China; ^2^School of Medicine, South China University of Technology, Guangzhou, China; ^3^Department of Biostatistics, School of Public Health, Brown University, Providence, RI, United States; ^4^Laboratory of Artificial Intelligence and 3D Technologies for Cardiovascular Diseases, Guangdong Provincial Key Laboratory of South China Structural Heart Disease, Guangdong Provincial People’s Hospital, Guangdong Academy of Medical Sciences, Guangzhou, China

**Keywords:** congenital aortic stenosis, pediatric aortic valve repair, aortic valve valvuloplasty, congenital heart disease, aortic valve morphology

## Abstract

**Background:**

Studies on the long-term outcomes of children with congenital aortic stenosis who underwent primary aortic repair are limited. We reviewed the long-term outcomes of children who underwent aortic valve (AoV) repair at our center.

**Methods:**

All children (*n* = 75) who underwent AoV repair between 2006 and 2020 were reviewed. The Kaplan-Meier curve was used to demonstrate the survival estimates. The Cox proportional hazard model and competing risk regression model were used to identify risk factors for death, reintervention, adverse events, and replacement.

**Results:**

The median age at surgery was 1.8 (IQR, 0.2–7.7) years, and the median weight at surgery was 10.0 (IQR, 5.0–24.0) kg. Early mortality and late mortality were 5.3% (4/75) and 5.6% (4/71), respectively. Risk factors for overall mortality were concomitant mitral stenosis (*P* = 0.01, HR: 9.8, 95% CI: 1.8–53.9), low AoV annulus *Z*-score (*P* = 0.01, HR: 0.6, 95% CI: 0.4–0.9), and prolonged cardiopulmonary bypass time (*P* < 0.01, HR: 9.5, 95% CI: 1.7–52.1). Freedom from reintervention was 72.9 ± 0.10% (95% CI: 56.3–94.4%) at 10 years. Risk factors for occurrence of adverse event on multivariable analysis included preoperative intubation (*P* = 0.016, HR: 1.004, 95% CI: 1.001–1.007) and a low AoV annulus *Z*-score (*P* = 0.019, HR: 0.714, 95% CI: 0.540–0.945). Tricuspid AoV morphology was associated with a suboptimal postoperative outcome (*P* = 0.03).

**Conclusion:**

Aortic valve repair remains a safe and durable solution for children with congenital aortic stenosis. Concomitant mitral stenosis and aortic valve anatomy, including tricuspid valve morphology and smaller annulus size, are associated with poor early and long-term outcomes.

## Introduction

Congenital aortic stenosis (CAS) is a lifelong disease, typically necessitating primary intervention during childhood and reintervention in later years (1). Treatments for CAS in children remain challenging, and each procedure have associated advantages and disadvantages. Considering the small aortic root and aortic valve (AoV) annulus, valve replacement is rarely performed as a preferred initial procedure. Mechanical AoV replacement requires lifelong anticoagulation and lacks growth potential. Additionally, studies have shown that the Ross procedure is not recommended as a primary intervention because of the high mortality in infants (2). It achieves higher freedom from autograft reintervention as an option for the second intervention than an option for the first intervention (3). As optimal primary treatments, surgical AoV repair and balloon aortic valvuloplasty provide similar long-term survival (4). However, several centers support that surgical AoV repair achieves better freedom from reintervention (5, 6).

Several studies have investigated the long-term outcomes in children who underwent AoV repair and have reported that the reintervention rate at 10 years was 27.9–47.9% (1, 7–10). Therefore, screening out the potential high-risk group for adverse events is essential for enhancing the follow-up of this group of patients and intervening in time. Most studies included patients who had undergone surgical aortic valvulotomy or balloon aortic valvuloplasty, which may impact prognosis after AoV repair. Furthermore, only one study used a competing risk regression model to analyze risk factors (7). We excluded patients who had undergone AoV intervention and used a competing risk regression model to determine the risk factors for reintervention and replacement. The prognostic effect of AoV number on patients with aortic stenosis remains controversial. Additionally, we explored the effect of valve leaflet morphologies on prognosis.

## Materials and methods

### Patients

**FIGURE 1:**
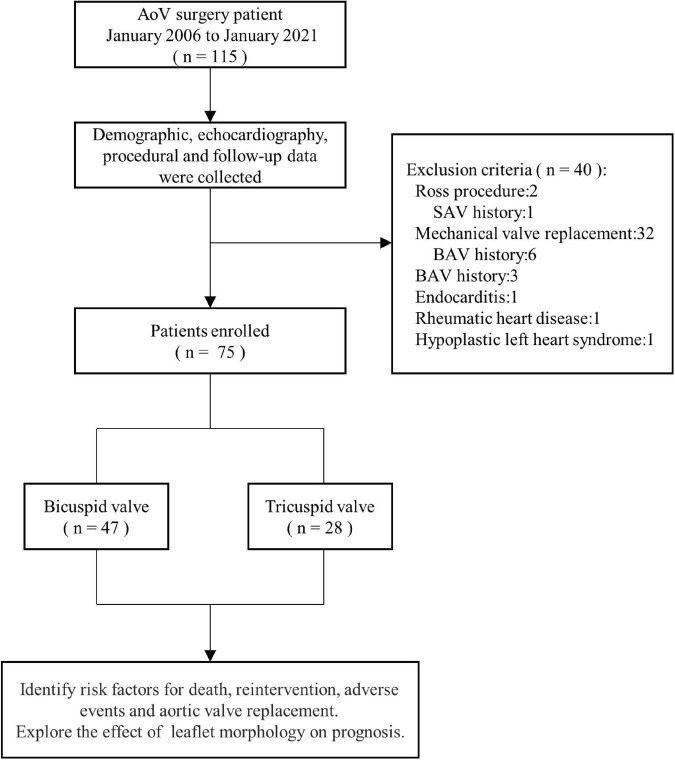
Study flow chart. AoV, aortic valve; BaV, balloon aortic valvuloplasty; SAV, surgical aortic valvulotomy.

From January 2006 to January 2021, 115 consecutive patients underwent AoV surgery at Guangdong Provincial People’s Hospital. The analysis excluded 27.8% (34/115) patients who underwent Ross operation or mechanical AoV replacement. Patients who had undergone balloon aortic valvuloplasty were excluded. Additionally, patients who had undergone surgical aortic valvotomy in other hospitals were excluded. Patients with endocarditis, rheumatic heart disease, or hypoplastic left heart syndrome were excluded from the analysis. Indications for surgery were AoV peak systolic gradient > 50 mmHg or AoV peak systolic gradient ≤ 50 mmHg with clinical symptoms. Medical records and echocardiography data were reviewed retrospectively until the most recent cardiology follow-up. The study flow chart is described in Figure 1.

### Definitions

Early mortality was defined as death occurring within 30 days of surgery or before discharge. All other deaths were considered late. Reinterventions were referred to as secondary AoV interventions, including balloon aortic valvuloplasty, surgical AoV repair, and AoV replacement. Any event involving death or reintervention was considered an adverse event.

Procedural success was defined by classifying outcomes into two groups (optimal and suboptimal) based on the final left ventricular outflow tract (LVOT) peak systolic gradient and aortic insufficiency (AI) grade following the procedure. These definitions are based on published articles and expert consensus (1, 11, 12). The optimal functional result consisted of a residual LVOT peak systolic gradient <35 mmHg and trivial or no AI. The suboptimal result was a residual LVOT peak systolic gradient ≥35 mmHg or mild or greater AI post-repair.

### Operative technique

Aortic valve repair was performed by median sternotomy in all patients. Following complete accession of AoV, commissurotomy was performed carefully to prevent AoV regurgitation. At least one of the following techniques were adopted for better valve mobility and function: resection of nodular dysplasia, thinning of valve leaflets, extension of cusp, and suspension of commissures.

### Data analysis

The endpoints were mortality, AoV reintervention, and replacement. Statistical analysis was performed using SPSS Statistics 25.0 (SPSS Inc., Chicago, IL, USA) and R software 4.2.0 (R Foundation, Beijing, China). Normally distributed continuous variables were reported as mean ±SD. Student’s *t* tests were used to compare differences between groups. For skewed continuous variables, the median [interquartile range (IQR)] was used to describe distributions, and the Wilcoxon-Mann-Whitney *U*-test was used to compare differences between groups. Descriptive statistics for categorical variables were reported as frequencies or percentages and compared using the Pearson χ^2^ or Fisher’s exact test. Peak AoV gradient before and after AoV repair were compared using paired *t* test. Time-related endpoints were analyzed and plotted using Kaplan-Meier actuarial survival. The Cox proportional hazard model determined the risk factors for death and adverse events. Reintervention, replacement, and death were not independent endpoints, as postoperative death may prevent later reintervention and replacement. Hence, a competing risk regression model was used to explore the risk factors for reintervention and replacement. All tests were two-tailed, and a *p*-value < 0.05 was considered statistically significant.

## Results

### Patient characteristics

A total of 75 patients [51 (68%) men; median age at surgery, (IQR, 0.2–7.7) year] were included in the study. Concomitant cardiovascular anomalies and valve morphologies are described in Table 1. Four patients underwent cardiac surgery before AoV repair (5.3%; 4/75). Previous cardiac surgery included ventricular septal defect (VSD) closure (*n* = 2), patent ductus arteriosus (PDA) ligation (*n* = 2), atrial septal defect (ASD) closure (*n* = 2), mitral valve (MV) repair (*n* = 1), and aortic arch repair for coarctation of the aorta (CoA, *n* = 1).

**TABLE 1:** Concomitant cardiovascular anomalies and aortic valve morphology in children who underwent AoV repair.

Abnormality	*N* (%)
PFO	18 (24.0)
PDA	15 (20.0)
CoA	12 (16.0)
Supravalvular aortic stenosis	10 (13.3)
ASD	8 (10.7)
VSD	7 (9.3)
Subaortic stenosis	6 (8.0)
Shone complex	1 (1.3)
Valvular abnormalities	
Tricuspid regurgitation	21 (28.0)
Mitral regurgitation	16 (21.3)
Mitral stenosis	5 (6.7)
Pulmonary stenosis	4 (5.3)
Pulmonary regurgitation	4 (5.3)
Aortic valve morphology	
Bicuspid	47 (62.7)
Tricuspid	28 (37.3)

PFO, patent foramen ovale; PDA, patent ductus arteriosus; CoA, coarctation of the aorta; ASD, atrial septal defect; VSD, ventricular septal defect.

Nine (12.0%, 9/75) patients underwent AoV repair with a patch to extend the free edges of the cusps. Forty-seven (62.7%; 47/75) patients had bicuspid valves, and others (37.3%; 28/75) had tricuspid valves. Concomitant cardiovascular anomalies were repaired in 48 (64%, 48/75) patients. The median aortic cross-clamp time and cardiopulmonary bypass time were 40 (IQR, 25–56) minutes and 76 (IQR, 52–104) minutes, respectively. Concomitant procedures included patent foramen ovale closure (*n* = 14), ASD closure (*n* = 4), PDA ligation (*n* = 15), CoA repair (*n* = 8), interrupted aortic arch repair (*n* = 1), VSD repair (*n* = 4), MV repair (*n* = 9), MV replacement (*n* = 1), supravalvular aortic stenosis resection (*n* = 8), subaortic stenosis resection (*n* = 9), pulmonary valve repair (*n* = 4), and temporary cardiac pacemaker implantation (*n* = 3). Baseline demographics, preoperative, intraoperative, and postoperative data are described in Table 2.

**TABLE 2:** Baseline characteristics.

Variable	Overall (*n* = 75)	Bicuspid valve (*n* = 47)	Tricuspid valve (*n* = 28)	*P*-value
Median age at operation	1.8 (0.2, 7.7)	2.8 (0.4, 8.2)	0.8 (0.2, 6.4)	0.32
Median weight at operation	10.0 (5.0, 24.0)	10.5 (6.0, 24.0)	7.0 (4.7, 23.5)	0.49
Concomitant repair	48 (64.0)	25 (53.2)	23 (82.1)	0.01
Median preoperative LVOT peak gradient, mm Hg	72.1 ± 20.3	75.5 ± 21.2	66.4 ± 17.6	0.06
Median ACC time, min	40.0 (25.0, 56.0)	35.0 (22.0, 54.0)	46.0 (32.5, 61.8)	0.03
Median CPB time, min	76.0 (52.0, 104.0)	67.0 (47.0, 100.0)	87.5 (64.8, 105.5)	0.01
Median postoperative LVOT peak gradient, mm Hg	30.5 ± 13.2	31.8 ± 13.0	27.8 ± 13.3	0.22
Postoperative moderate or greater AI	6 (8.0)	5 (10.6)	1 (4.5)	0.02
Optimal postoperative outcome	32 (42.7)	27 (57.4)	5 (19.2)	0.03
Intubation time, h	17.5 (6.5, 97.5)	19.0 (6.0, 73.5)	13.8 (7.1, 220.9)	0.35
ICU stay, day	2 (1,5)	1.5 (1.0, 3.0)	2 (1.0, 9.4)	0.81
Death	8 (10.6)	4 (8.5)	4 (14.3)	0.46
AoV reoperation	8 (11.3%)	5 (10.6)	3 (10.7)	> 0.99

LVOT, Left ventricular outflow tract; ACC, aortic crossclamp; CPB, cardiopulmonary bypass; AI, aortic insufficiency; ICU, intensive care unit; AoV, aortic valve.

### Aortic valve function

Immediate postoperative echocardiographic data were unavailable for two (2.7%, 2/75) patients. The mean LVOT peak gradient for the whole cohort decreased from 72.1 ± 20.3 mmHg to 30.5 ± 13.2 mmHg (*P* < 0.01). AI progressed in 13 (17.3%, 13/75) patients, of whom nine (12.0%, 9/75) patients had mild regurgitation without need for intervention, two (2.7%, 2/75) patients had moderate regurgitation, and two (2.7%, 2/75) patients had severe regurgitation. The outcome was optimal in 32 (43.8%) patients and suboptimal in 41 (56.2%) patients.

### Mortality

**FIGURE 2:**
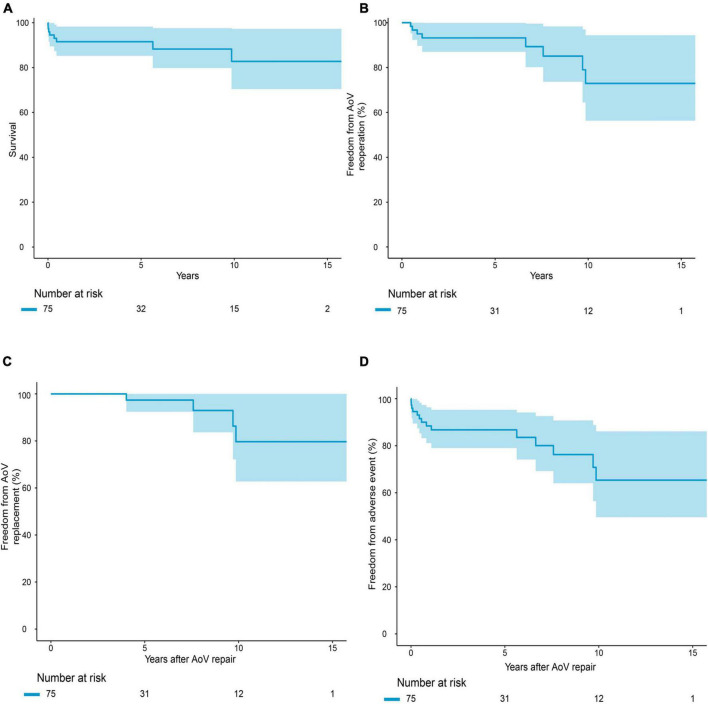
Survival **(A)** freedom from reoperation **(B)** Adverse event **(C)** Replacement **(D)** for the overall cohort.

**FIGURE 3:**
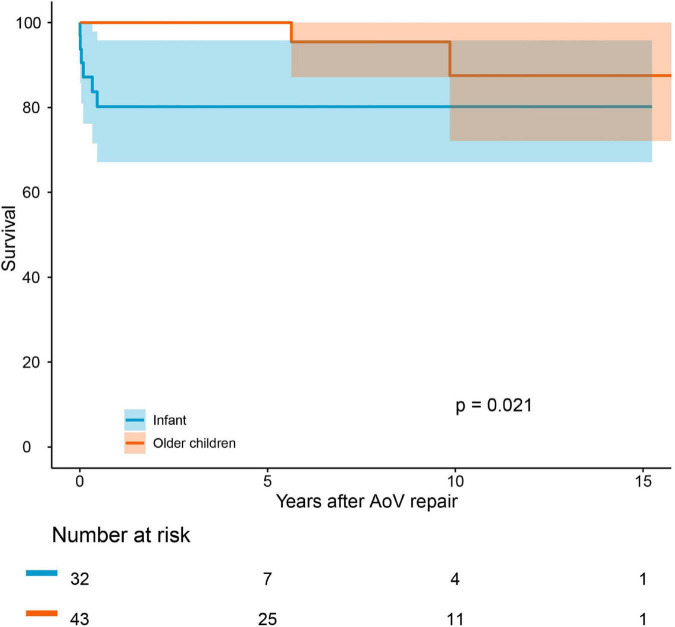
Survival comparing of infants and children older than 1 year of age.

**FIGURE 4:**
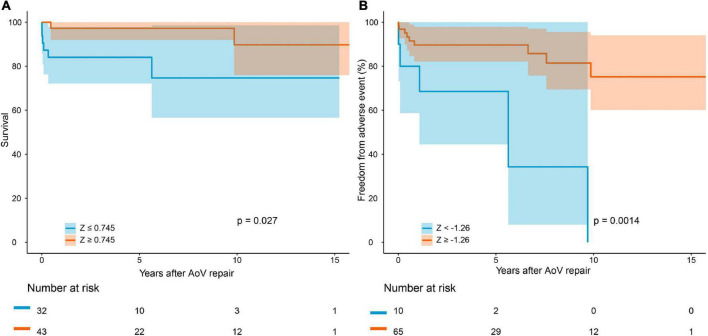
Survival after AoV repair **(A)** and freedom from adverse event **(B)** in patients with different aortic annulus diameter *Z*-score.

The median follow-up period was 4.1 years. Follow-up was completed in 95.7% of the survivors. The causes of death are summarized in Supplementary Table 1. Early mortality and late mortality were 5.3% (4/75) and 5.6% (4/71), respectively. Overall survival was 91.5 ± 0.03% (95% CI: 85.3–98.3%) at 5 years and 82.7 ± 0.07% (95% CI: 70.4–97.3%) at 10 years (Figure 2A). In older children, survival was 100% at 5 years and 87.5 ± 0.09% (95% CI: 72.1–99.9%) at 10 years. Survival was lower in infants than in older children (*P* = 0.021, Figure 3). Risk factors for overall mortality on multivariable analysis included concomitant mitral stenosis (*p* = 0.01, HR: 9.8, 95% CI: 1.8–53.9), low AoV annulus *Z*-score (*P* = 0.01, HR: 0.6, 95% CI: 0.4–0.9), and prolonged cardiopulmonary bypass (CPB) time (*P* < 0.01, HR: 9.5, 95% CI: 1.7–52.1) (Table 3). The receiver operating characteristic curve and Youden J index analysis identified a cutoff value for AoV annulus diameter *Z*-score of 0.745 (sensitivity, 61.2%; specificity, 75%; area under the curve, 0.69). This cutoff value appropriately categorized survival when applied to our study cohort (Figure 4A).

**TABLE 3:** Univariate and multivariate analyses for overall death.

Variable	Univariable			Multivariable		
		
	*P*-value	Hazard ratio	95% CI	*P*-value	Hazard ratio	95% CI
Infant	0.039	0.180	0.036–0.915			
Preoperative intubation	0.050	4.220	0.999–17.818			
MS	0.001	10.509	2.477–44.588	0.009	9.763	1.767–53.937
LVOT or MS	0.022	6.617	1.316–33.257			
AoV annulus Z-score	0.026	0.651	0.469–0.903	0.011	0.600	0.406–0.887
LVPWD	0.074	0.722	0.506–1.032			
CPB	< 0.001	1.019	1.009–1.030	0.001	1.020	1.008–1.032
Postoperative-intubation time	0.007	1.004	1.001–1.007			

MS, mitral stenosis; LVOT, left ventricular outflow tract; LVPWD, left ventricular posterior wall dimension; CPB, cardiopulmonary bypass.

### Reintervention

Eight (11.3%, 8/71) patients required reintervention. The reasons for reintervention and reintervention type are described in Supplementary Table 2. AoV reintervention occurred at a median of 3.9 (range, 0.4–9.7) years after primary repair. Four patients underwent AoV replacement. AoV replacement was performed at a median of 8.6 (range, 4.2–9.9) years after primary repair.

Overall freedom from any reintervention was 93.2 ± 0.03% (95% CI: 87.0–99.9%) at 5 years and 72.9 ± 0.10% (95% CI: 56.3–94.4%) at 10 years (Figure 2B). The competing risk model showed that preoperative moderate or greater AI (*P* = 0.048, HR: 6.3, 95% CI: 1.0–39.5) was the risk factor associated with reintervention (Table 4). Freedom from AoV replacement was 97.4 ± 0.03% (95% CI: 92.4–99.9%) at 5 years and 79.7 ± 0.10% (95% CI: 62.8–99.9%) at 10 years (Figure 2C). Competing risk models showed that postoperative moderate or greater AI (*P* = 0.030, HR: 10.4, 95% CI: 1.3–85.8) and weight at surgery (*P* < 0.001, HR: 6.3, 95% CI: 1.0–1.1) (Table 4) were risk factors associated with reintervention.

**TABLE 4:** Risk factors for reintervention and replacement by competing risk regression model.

Variable	*P*-value	Hazard ratio	95.0% CI	
			
			Lower	Upper
**Reintervention**				
Preoperative significant AI	0.048	6.332	1.014	39.533
Concomitant operation	0.071	0.123	0.013	1.193
Postoperative – significant AI	0.109	8.138	0.629	105.343
**Replacement**				
Weight	< 0.001	1.048	1.021	1.076
Preoperative -LVEDD *Z*-score	0.810	0.949	0.618	1.456
Preoperative -PAG	0.273	0.967	0.911	1.027
Postoperative significant AI	0.030	10.373	1.254	85.833

AI, aortic insufficiency; LVEDD, left ventricular end diastolic diameter; PAG, peak aortic gradient.

### Adverse event

Overall freedom from any adverse event was 86.8 ± 0.04% (95% CI: 79.0–95.3%) at 5 years and 65.4 ± 0.09% (95% CI: 49.6–86.1%) at 10 years (Figure 2D). In older children, freedom from adverse events was higher than in infants (*P* = 0.07). Risk factors for occurrence of adverse events on multivariable analysis included preoperative intubation (*P* = 0.016, HR: 1.004, 95% CI: 1.001–1.007) and a low AoV annulus *Z*-score (*P* = 0.019, HR: 0.714, 95% CI: 0.540–0.945) (Table 5). The cutoff value for the AoV annulus diameter *Z*-score was –1.26 (sensitivity, 91.8%; specificity, 35.7%; area under the curve, 0.66) (Figure 4B).

**TABLE 5:** Univariate and multivariate analyses for adverse event.

Variable	Univariable			Multivariable		
		
	*P*-value	Hazard ratio	95% CI	*P*-value	Hazard ratio	95% CI
Infant	0.081	0.384	0.131–1.126			
sex	0.071	6.535	0.854–49.989			
Preoperative intubation	0.074	2.895	0.902–9.287	0.016	1.004	1.001–1.007
MS	0.013	2.481	0.893–6.892			
AoV annulus *Z*-score	0.014	0.697	0.522–0.930	0.048	0.709	0.505–0.996
Post-AI	0.027	4.506	1.192–17.043			
CPB	< 0.001	1.016	1.008–1.024			
Postoperative-intubation time	0.003	1.004	1.002–1.007			

MS, mitral stenosis; AI, aortic insufficiency; CPB, cardiopulmonary bypass.

### Outcomes according to aortic valve morphology

Patients with tricuspid AoV morphology were at higher risk of suboptimal outcomes (*P* = 0.03). Although the incidence of concomitant repair and suboptimal outcome was higher in patients with tricuspid AoV morphology, no significant differences in postoperative intubation time (*P* = 0.35) and ICU duration (*P* = 0.81) existed between the two groups. No differences in survival (*P* = 0.36), freedom from reintervention (*P* = 0.61), and adverse event (*P* = 0.14) existed between patients with tricuspid and bicuspid AoV morphologies.

## Discussion

Our study elicited three main findings. First, AoV repair was a successful primary intervention and postponed the time of reoperation or AoV replacement to a suitable age. Second, concomitant MS, low AoV annulus *Z*-score, and prolonged cardiopulmonary bypass time were risk factors for death. Preoperative intubation and a low AoV annulus *Z*-score were risk factors for adverse events. Third, tricuspid AoV morphology was associated with higher incidence of suboptimal outcomes than bicuspid AoV morphology.

In the past decades, balloon aortic valvuloplasty (BAV) was popular in several centers. However, balancing the residual peak aortic gradient and postoperative AI is challenging, because it may break the weakest, thinnest part of the leaflet and cannot relieve the stenosis directly and precisely as AoV repair would (5, 6). With development of new surgical techniques, including leaflet thinning, commissural suspension, and cusp extension, AoV repair currently achieves a better long-term outcome in children with CAS (1, 13, 14), including neonates and infants (6, 15). Therefore, our center has preferred AoV as the initial AoV repair in recent years.

### Mortality

In our study, early mortality and late mortality were 5.3% (4/75) and 5.6% (4/71), respectively. Similarly, Schlein et al. (7) reported that early mortality and late mortality were 5.6 and 6.7%, respectively. Furthermore, in patients with isolated AS, survival was much higher. Wallace et al. (1) reported early mortality of 0.9% and late mortality of 0.9% in their cohort of isolated patients with AS who underwent AoV repair.

Additionally, we found that the presence of mitral stenosis is an independent risk factor for death. Similarly, the presence of multiple left-heart obstructions was associated with mortality in this study. Brown et al. (16) found that most patients with multiple left-heart obstructions had pulmonary hypertension, which is associated with mortality. Concurrently, multiple left-heart obstructions lead to impairment of global cardiac function, which may increase the risk of death. The subset of patients is diagnosed with incomplete shone syndrome (17), and their operative mortality is adversely affected by the severity of mitral valve disease (18).

### Reintervention and adverse event

Freedom from reintervention, ranging from 35 to 78% after 10 years of follow-up, remains a vexing surgical problem (8, 19–21). In the overall cohort, freedom from reintervention and replacement at 10 years was 72.9 and 79.9%, respectively. The mean age at reintervention was 9.3 years, when the Ross procedure could achieve excellent outcomes. Ross procedure achieves higher freedom from autograft reintervention as an option for the second intervention than an option for the first intervention (3). Therefore, AoV repair achieves durable and acceptable freedom reintervention as a primary intervention.

In accordance with the previous report (7, 8, 22), we showed that a small aortic annulus was associated with mortality and adverse event. Although aortic annulus size is not a risk factor for reintervention in the competing risk model, we considered it to imply a poor outcome. A diminished aortic annulus offers less room or possibility for repair and increases the difficulty of surgery. Concurrently, a small annulus predicts a higher pressure gradient after surgery because it cannot be relieved by repair surgery, which can explain the increased risk of mortality and reintervention. Similarly, Reich et al. (23) demonstrated that hypoplastic annulus increases the risk of both BAV and AoV repair. Additionally, Schlein et al. argued that aortic annulus size and mitral valve condition determine the prognosis for patients who underwent AoV repair (7, 8). Our study demonstrated that primary AoV repair achieves superior survival in patients with an AoV annulus diameter *Z*-score ≥0.745 and freedom from adverse event in patients with an AoV annulus diameter *Z*-score≥–1.26. A further study is required to determine the optimal approach for patients with smaller AoV annulus.

Furthermore, this study confirmed that preoperative moderate or greater AI was an independent risk factor for reintervention and postoperative AI for replacement in the competing risk regression model. Auld et al. (24) found that postoperative AI could predict worse freedom from intervention. Other previous studies demonstrated that patients with postoperative AI were likely to require early AoV replacement (24, 25), which can be explained by the fact that volume overload is more difficult for adjustment by the left ventricular myocardium than pressure overload (5). Furthermore, repair for individuals with combined AS and AI is more challenging. Therefore, AoV replacement is the preferred resolution.

### Aortic valve morphology

The effect of AoV morphology on long-term outcome remains controversial. In our study, we found no difference in long-term outcomes between children with tricuspid valves and those with bicuspid valves. Vergnat et al. (15) suggested that neonates with tricuspid arrangement after open valvoplasty achieve higher freedom from replacement. Conversely, Wallace et al. (1) showed that tricuspid AoV was associated with worse freedom from reintervention and postoperative suboptimal outcome, which implied lower freedom from reoperation. Although we found no association between reoperation and AoV morphology due to limited sample size and follow-up time, tricuspid AoV morphology was associated with a suboptimal outcome. This finding may be due to the complex techniques used to achieve tricuspidization (26). Further studies and longer follow-up times are required to determine whether AoV morphology affects reoperation or AoV replacement.

## Limitations

This study had several limitations. First, this retrospective study included a limited number of patients who underwent AoV repair in a single center for 15 years. Second, we could not preclude results from being influenced by differences between surgeons and perform subgroup analysis by surgeons because of the limited number of patients.

## Conclusion

Our results support that AoV repair for patients with CAS can be a successful primary strategy. The independent predictors of death were concomitant mitral stenosis, smaller AoV annulus, and prolonged CPB time. Patients with low AoV annulus *Z*-score and preoperative intubation were at higher risk of adverse event. The incidence of the suboptimal operative outcome in patients with tricuspid valve morphology was higher than in patients with bicuspid valve morphology.

## Data availability statement

The raw data supporting the conclusions of this article will be made available by the authors, without undue reservation.

## Ethics statement

The study was approved by the Guangdong Provincial People’s Hospital Institutional Research Ethics Board (2019338H).

## Author contributions

QR, JY, and TC wrote the manuscript and conducted the statistical analysis. HQ, EJ, and TL inspected and validated the data. JZ and XL provided the funding support and supervision. SW, JC, and XX revised the manuscript. All authors contributed to the article and approved the submitted version.

## References

[1] Wallace F Buratto E Schulz A D’Udekem Y Weintraub RG Brizard CP Long-term outcomes of primary aortic valve repair for isolated congenital aortic stenosis in children. *J Thorac Cardiovasc Surg.* (2022): [Epub ahead of print]. 10.1016/j.jtcvs.2021.11.097 35430079

[2] Buratto E Konstantinov IE. Aortic valve surgery in children. *J Thorac Cardiovasc Surg.* (2020) 161:244–50. 10.1016/j.jtcvs.2020.06.145 32891449

[3] Buratto E Wallace F Fricke TA Brink J D’Udekem Y Brizard CP Ross procedures in children with previous aortic valve surgery. *J Am Coll Cardiol.* (2020) 76:1564–73. 10.1016/j.jacc.2020.07.058 32972534

[4] Hill GD Ginde S Rios R Frommelt PC Hill KD. Surgical valvotomy versus balloon valvuloplasty for congenital aortic valve stenosis: a systematic review and meta-analysis. *J Am Heart Assoc.* (2016) 5:e003931. 10.1161/JAHA.116.003931 27503847 PMC5015309

[5] Brown JW Rodefeld MD Ruzmetov M Eltayeb O Yurdakok O Turrentine MW Surgical valvuloplasty versus balloon aortic dilation for congenital aortic stenosis: are evidence-based outcomes relevant? *Ann Thorac Surg.* (2012) 94:146–53, discussion 153–155. 10.1016/j.athoracsur.2012.02.054 22537535

[6] Siddiqui J Brizard CP Galati JC Iyengar AJ Hutchinson D Konstantinov IE Surgical valvotomy and repair for neonatal and infant congenital aortic stenosis achieves better results than interventional catheterization. *J Am Coll Cardiol.* (2013) 62:2134–40. 10.1016/j.jacc.2013.07.052 23954309

[7] Schlein J Kaider A Gabriel H Wiedemann D Hornykewycz S Simon P Aortic valve repair in pediatric patients - 30 years single center experience. *Ann Thorac Surg.* (2022): [Epub ahead of print]. 10.1016/j.athoracsur.2022.05.061 35779601

[8] Galoin-Bertail C Capderou A Belli E Houyel L. The mid-term outcome of primary open valvotomy for critical aortic stenosis in early infancy - a retrospective single center study over 18 years. *J Cardiothorac Surg.* (2016) 11:116. 10.1186/s13019-016-0509-9 27484000 PMC4970304

[9] Poncelet AJ El Khoury G De Kerchove L Sluysmans T Moniotte S Momeni M Aortic valve repair in the paediatric population: insights from a 38-year single-centre experience. *Eur J Cardiothorac Surg.* (2017) 51:43–9. 10.1093/ejcts/ezw259 27681035

[10] Vergnat M Asfour B Arenz C Suchowerskyj P Bierbach B Schindler E Contemporary results of aortic valve repair for congenital disease: lessons for management and staged strategy. *Eur J Cardiothorac Surg.* (2017) 52:581–7. 10.1093/ejcts/ezx172 28874025

[11] Boe BA Zampi JD Kennedy KF Jayaram N Porras D Foerster SR Acute success of balloon aortic valvuloplasty in the current era: a national cardiovascular data registry study. *JACC Cardiovasc Interv.* (2017) 10:1717–26. 10.1016/j.jcin.2017.08.001 28882282

[12] Torres A Vincent JA Everett A Lim S Foerster SR Marshall AC Balloon valvuloplasty for congenital aortic stenosis: multi-center safety and efficacy outcome assessment. *Catheter Cardiovasc Interv.* (2015) 86:808–20. 10.1002/ccd.25969 26032565

[13] D’Udekem Y Siddiqui J Seaman CS Konstantinov IE Galati JC Cheung MM Long-term results of a strategy of aortic valve repair in the pediatric population. *J Thorac Cardiovasc Surg.* (2013) 145:461–9. 10.1016/j.jtcvs.2012.11.033 23246057

[14] Kari FA Kroll J Kiss J Hess C Stiller B Siepe M Progression of aortic regurgitation after different repair techniques for congenital aortic valve stenosis. *Pediatr Cardiol.* (2016) 37:84–9. 10.1007/s00246-015-1243-0 26266328

[15] Vergnat M Asfour B Arenz C Suchowerskyj P Bierbach B Schindler E Aortic stenosis of the neonate: a single-center experience. *J Thorac Cardiovasc Surg.* (2019) 157:318–26. 10.1016/j.jtcvs.2018.08.089 30557949

[16] Brown DW Dipilato AE Chong EC Gauvreau K McElhinney DB Colan SD Sudden unexpected death after balloon valvuloplasty for congenital aortic stenosis. *J Am Coll Cardiol.* (2010) 56:1939–46. 10.1016/j.jacc.2010.06.048 21109118

[17] Aslam S Khairy P Shohoudi A Mercier LA Dore A Marcotte F Shone complex: an under-recognized congenital heart disease with substantial morbidity in adulthood. *Can J Cardiol.* (2017) 33:253–9. 10.1016/j.cjca.2016.09.005 27956040

[18] Brown JW Ruzmetov M Vijay P Hoyer MH Girod D Rodefeld MD Operative results and outcomes in children with Shone’s anomaly. *Ann Thorac Surg.* (2005) 79:1358–65. 10.1016/j.athoracsur.2004.09.013 15797077

[19] Miyamoto T Sinzobahamvya N Wetter J Kallenberg R Brecher AM Asfour B Twenty years experience of surgical aortic valvotomy for critical aortic stenosis in early infancy. *Eur J Cardiothorac Surg.* (2006) 30:35–40. 10.1016/j.ejcts.2006.03.050 16725339

[20] Hraška V Sinzobahamvya N Haun C Photiadis J Arenz C Schneider M The long-term outcome of open valvotomy for critical aortic stenosis in neonates. *Ann Thorac Surg.* (2012) 94:1519–26. 10.1016/j.athoracsur.2012.03.056 22607784

[21] Polimenakos AC Sathanandam S Elzein C Barth MJ Higgins RS Ilbawi MN. Aortic cusp extension valvuloplasty with or without tricuspidization in children and adolescents: long-term results and freedom from aortic valve replacement. *J Thorac Cardiovasc Surg.* (2010) 139:933–41, discussion 941. 10.1016/j.jtcvs.2009.12.015 20304137

[22] Agnoletti G Raisky O Boudjemline Y Ou P Bonnet D Sidi D Neonatal surgical aortic commissurotomy: predictors of outcome and long-term results. *Ann Thorac Surg.* (2006) 82:1585–92. 10.1016/j.athoracsur.2006.05.049 17062209

[23] Reich O Tax P Marek J Razek V Gilik J Tomek V Long term results of percutaneous balloon valvoplasty of congenital aortic stenosis: independent predictors of outcome. *Heart.* (2004) 90:70–6. 10.1136/heart.90.1.70 14676248 PMC1768014

[24] Auld B Carrigan L Ward C Justo R Alphonso N Anderson B. Balloon aortic valvuloplasty for congenital aortic stenosis: a 14-year single centre review. *Heart Lung Circ.* (2019) 28:632–6. 10.1016/j.hlc.2018.02.014 29625867

[25] Karamlou T Shen I Alsoufia B Burch G Reller M Silberbach M The influence of valve physiology on outcome following aortic valvotomy for congenital bicuspid valve in children: 30-year results from a single institution. *Eur J Cardiothorac Surg.* (2005) 27:81–5. 10.1016/j.ejcts.2004.10.044 15621475

[26] Schulz A Buratto E Wallace F Fulkoski N Weintraub RG Brizard CP Outcomes of aortic valve repair in children resulting in bicuspid anatomy: is there a need for tricuspidization? *J Thorac Cardiovasc Surg.* (2022) 164:186–96. 10.1016/j.jtcvs.2022.01.022 35227498

