# Supplementary material for: Long-term outcomes of primary aortic valve repair in children with congenital aortic stenosis – 15-year experience at a single center
Source: Front Cardiovasc Med. 2022 Oct 13;9:1029245. doi: 10.3389/fcvm.2022.1029245 (PMC9613358; doi:10.3389/fcvm.2022.1029245)
Supplement: Supplementary file 1 [file Data_Sheet_1.docx]

Supplementary Material

**Supplementary material**

Table E1.Description of death following AoV repair

| **Age** | **Year of operation** | **Weight** | **Cardiac comorbidities** | **Concomitant operation** | **Death**  **(year)** | **Cause of death** |
| --- | --- | --- | --- | --- | --- | --- |
| 10 months | 2020 | 6.4kg | Mitral stenosis and regurgitation  Pulmonary valve regurgitation  Tricuspid regurgitation | LVOT reconstruction  MV replacement | Early | Failure wean off ECMO,  Decesion to withdraw treatment |
| 14 days | 2017 | 3.2kg | VSD,PDA,PFO  Coarctation of the aorta  Tricuspid regurgitation  Mitral regurgitation  Pulmonary valve regurgitation | VSD closure  PDA ligation | Early | Cardiac arrest  ECMO declined |
| 5 months | 2015 | 4.3kg | ASD,PDA  Aortic arch hypoplasia  Mitral stenosis and regurgitation | PDA ligation  Aortic arch repair | Early | Multi-organ failure |
| 9 months | 2014 | 7kg | supravalvular aortic stenosis  Tricuspid regurgitation  Mitral regurgitation | Resection of supravalvular aortic stenosis | Early | Failure to wean off bypass.  ECMO declined |
| 2 months | 2015 | 4.5kg | PFO  Tricuspid stenosis and regurgitation  Mitral stenosis and regurgitation  Pulmonary valve stenosis and regurgitation | Pulmonary valve repair  Resection of subaortic stenosis | Late  (3 months) | Sudden arrhythmia after discharge |
| 2 months | 2011 | 5.1kg | PFO  Mitral regurgitation | Nil | Late  (6 months) | Subarachnoid hemorrhage after reoperation by BAV |
| 4 years | 2011 | 15.5kg | supravalvular aortic stenosis  Mitral regurgitation  Pulmonary valve stenosis | MV repair  Pulmonary repair  Supravalvular aortic stenosis repair | Late  (5.5 years) | Unknown aetiology |
| 14 years | 2011 | 40kg | Nil | Nil | Late  (10 years) | Infective endocarditis |

Table E2.Description of first reintervention following AoV repair

| **Age** | **Year of operation** | **Weight** | **Cardiac comorbidities** | **Primary**  **operation** | **Time**  **(year)** | **reintervention** | **Cause** |
| --- | --- | --- | --- | --- | --- | --- | --- |
| 0.14 | 2011 | 5.1 | PFO  Mitral regurgitation | Commissurotomy | 0.4 | BAV | AS |
| 9.32 | 2010 | 46 | Tricuspid regurgitation | Commissurotomy | 0.6 | BAV | AS |
| 4.65 | 2019 | 19 | VSD,PDA  Tricuspid regurgitation  Mitral regurgitation | Commissurotomy  Commissural suspension  VSD closure  PDA ligation | 0.8 | Cusp extension | AI |
| 0.56 | 2017 | 7 | Mitral regurgitation | Commissurotomy  Resection of nodular dysplasia  MV repair | 1.1 | Commissurotomy  Leaflet thinning | AS |
| 1.41 | 2013 | 10.5 | Nil | Commissurotomy  Cusp extension | 6.6 | BAV | AS |
| 7.41 | 2008 | 20 | Coarctation of the aorta  Mitral regurgitation | Commissurotomy | 7.5 | Mechanical valve replacement | Unkown  (external institution) |
| 0.21 | 2006 | 5 | Interrupted aortic arch  Tricuspid regurgitation | Commissurotomy  Aortic arch repair | 9.6 | Mechanical valve replacement  Supravalvular aortic stenosis repair | AS  AI |
| 14.26 | 2011 | 40 | Nil | Commissurotomy | 9.7 | Bentall | IE |
